# A maize polygalacturonase functions as a suppressor of programmed cell death in plants

**DOI:** 10.1186/s12870-019-1897-5

**Published:** 2019-07-15

**Authors:** Yijian He, Shailesh Karre, Gurmukh S. Johal, Shawn A. Christensen, Peter Balint-Kurti

**Affiliations:** 1 Dept. of Entomology and Plant Pathology, NC State University, Raleigh, NC 27695-7616 USA; 2 Botany and Plant Pathology, Purdue University, West Lafayette, USA; 3 Chemistry Research Unit, Center for Medical, Agricultural, and Veterinary Entomology, Department of Agriculture–Agricultural Research Service (USDA–ARS), Gainesville, FL 32608 USA; 4 Plant Science Research Unit, USDA-ARS, NC State University, Raleigh, NC 27695-7616 USA

**Keywords:** Hypersensitive response, Maize, Polygalacturonase

## Abstract

**Background:**

The hypersensitive defense response (HR) in plants is a fast, localized necrotic response around the point of pathogen ingress. HR is usually triggered by a pathogen recognition event mediated by a nucleotide-binding site, leucine-rich repeat (NLR) protein. The autoactive maize NLR gene *Rp1-D21* confers a spontaneous HR response in the absence of pathogen recognition. Previous work identified a set of loci associated with variation in the strength of *Rp1-D21*-induced HR. A polygalacturonase gene homolog, here termed *ZmPGH1,* was identified as a possible causal gene at one of these loci on chromosome 7.

**Results:**

Expression of *ZmPGH1* inhibited the HR-inducing activity of both *Rp1-D21* and that of another autoactive NLR, RPM1(D505V), in a *Nicotiana benthamiana* transient expression assay system. Overexpression of *ZmPGH1* in a transposon insertion line of maize was associated with suppression of chemically-induced programmed cell death and with suppression of HR induced by *Rp1-D21* in maize plants grown in the field.

**Conclusions:**

*ZmPGH1* functions as a suppressor of programmed cell death induced by at least two autoactive NLR proteins and by two chemical inducers. These findings deepen our understanding of the control of the HR in plants.

**Electronic supplementary material:**

The online version of this article (10.1186/s12870-019-1897-5) contains supplementary material, which is available to authorized users.

## Background

The plant innate immune system is often thought of a two inter-related systems [1]. The first uses membrane-bound pattern recognition receptors (PRRs) to detect microbial “marker” molecules often called pathogen- or microbe-associated molecular patterns (PAMPs or MAMPs). This recognition triggers the multi-faceted, relatively low-level, MAMP-triggered immunity (MTI) response, also known as basal defense [2]. A class of pathogen-derived proteins known as effectors are delivered into plant cells and aid the pathogenesis process. Some of them target host proteins to suppress MTI [3]. Some are directly or indirectly recognized by plant resistance (R-) proteins. This recognition triggers the effector-triggered immunity (ETI) defense response [2]. ETI is faster and stronger than MTI though qualitatively the two response are similar in many respects [4, 5]. ETI responses usually include a fast, localized necrosis at the point of initial pathogen ingress, called the hypersensitive response (HR) which is a form of programed cell death (PCD). Most R-genes encode proteins with nucleotide binding and leucine-rich repeat domains (NLRs) and have coiled coil or toll-interleukin receptor (CC or TIR) domains at the N-terminus (respectively CNLs and TNLs) [6]. Inappropriate activation of NLRs can lead to spontaneous cell death and growth and yield penalties [7, 8].

The complex maize *Rp1* locus carries a number of CNL R-gene paralogs that are tandemly-repeated and that have the capacity to recognize and mediate resistance to certain races of the fungus causing common rust, *Puccinia sorghi* [9]. Due to its repetitive nature, unequal crossover events at this locus are frequently observed [10]. In some cases these events lead to intragenic recombination, and the production of a chimeric gene. The gene *Rp1-D21* resulted from recombination between two *Rp1* genes, *Rp1-D* and *Rp1-dp2*. The Rp1-D21 protein is spontaneously activated and causes HR on leaves of uninfected maize plants [11, 12]. The strength of HR conferred Rp1-D21, as well as other associated phenotypes such as stunted growth are dependent on genetic background [13].

To identify loci and gene candidates associated with modifying *Rp1-D21-*mediated spontaneous HR, and possibly HR in general, we previously undertook a large mapping project. We crossed *Rp1-D21* into more than 3600 of the lines of the maize NAM population [14] and performed a detailed genome-wide nested association mapping study to identify 44 single nucleotide polymorphism (SNP) loci associated with variation in the spontaneous HR phenotype [15]. While the closest predicted gene at each locus was most likely to be causal (the candidate gene), further validation was required to establish causality. In previous work, we demonstrated causality for two predicted lignin biosynthesis pathway genes [16, 17] and one candidate predicted to be a component of the mitochondrial electron transport chain [18].

**Fig. 1:**
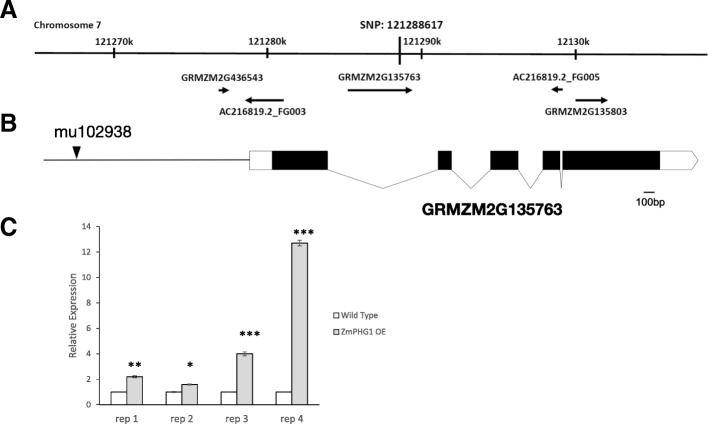
The mu1029380 transposon inserted at the upstream of *ZmPGH1* gene causes enhanced expression. (**a**) The genes near the SNP at 121,288,617 bp on chromosome 7 (based on the maize B73 genome v2 at maizegdb.org), which was associated with variation in Rp1-D21-induced HR (Olukolu, Wang et al. 2014). Arrows indicate the direction and length of transcription of the genes; (**b**) The position of mu1029380 insertion relative to *ZmPGH1* gene. White boxes indicate the UTRs. Black boxes indicate the exons. The polylines indicate the introns. The scale bar of this figure is 100 bp; (**c**) By qRT-PCR, we found the expression of *ZmPGH1* gene was enhanced in four F_2_ segregants from the UFMu-02561 family homozygous for the mu1029380 insertion (here termed ZmPGH1OE plants) compared to siblings homozygous for the absence of the insertion (termed wild type plants). Figure shows the result of one biological replication pooled from four plants. Three technical replications were performed for each biological replication. Error bars represent standard deviation. All replications demonstrated at least 1.6 fold enhanced expression of *ZmPGH1* gene in ZmPGH1OE plants. Significant difference from wild type at * *p* < 0.1 ** *p* < 0.05 ***p < 0.01 ****p < 0.001. Significance determined by T-test

Another highly-associated SNP was located at 121,288,617 bp on chromosome 7 (Fig. 1a, based on the maize B73 genome v2 at maizegdb.org), in the coding sequence of gene GRMZM2G135763 which encoded a gene with high homology to polygalacturonase. The expression level of GRMZM2G135763 was enhanced about 2-fold by Rp1-D21-induced HR in two different genetic backgrounds [15]. The work presented here was undertaken with the goal of determining whether this gene was actually underlying variation in HR caused by Rp1-D21.

## Methods

### Plant materials and growth conditions

Maize plants used for cell death and RT-qPCR assays were raised in chambers in the NCSU Phytotron with the following conditions: 16 h light/8 h dark photoperiod, day/night temperature 25 °C/18 °C. Field disease evaluations were undertaken at Central Crops Research Station (Clayton, North Carolina, USA). Twelved kernels of were sown in 1.8 m rows with a 0.6 m inter-row spacing. *Nicotiana benthamiana* plants were grown at 23 °C with a 16 h light/8 h dark photoperiod.

The UniformMu insertion mutant line UFMu-02561 in the background of the W22 maize line and the W22 line itself were received from Maize Genetics Cooperation Stock Center (IL, USA). The mu1029380 Mu insertion was genotyped by PCR [19]. The sequences of genotyping primers are provided in Text S1.

For field evaluation of the effect of the mu1029380 Mu insertion on the Rp1-D21 phenotype we evaluated a population derived from a series of crosses between UFMu-02561 and W22:Rp1-D21 – a W22 inbred line into which the Rp1-D21 gene had been introgressed through repeated backcrossing - in which the mu1029380 insertion was segregating 1:1 homozygous:heterozygous and the Rp1-D21 gene was also segregating 1:1 heterozygous:absent. Populations were evaluated in summer 2018 at Clayton, NC, USA. The experiment was conducted using two replications and a randomized complete block design with. Each replicate had 48 plants, grown in four rows with twelve plants per row. W22:Rp1-D21 was generated in Purdue by Dr. Johal.

### Plasmid constructs

The 35S::HCT1806-EGFP and 35S::Rp1-D21-HA plasmids were previously described [16]. The GRMZM2G135763 coding sequence (called here *ZmPGH1*) without its stop-codons were PCR-amplified. The coding sequence of β-glucuronidase (GUS) was previously published [16]. The plasmid expressing RPM1(D505V) was obtained from Dr. Jeff Dangl and has been described previously [16]. *ZmPGH1* and *Gus* were introduced into pGWB641 vector (35S promoter and C-terminal EYFP tag) [20] using the Gateway LR recombination reaction. For the agrobacterium-mediated transient assay, the destination plasmids were transformed into the GV3101 agrobacterium strain using heat shock transformation. Primer sequences are provided in Additional file 1: Table S1.

### Transient assay

These methods for the *N. benthamiana Agrobacterium* transient assay have been described previously [17, 18].

### Chemical-induced cell death assay

This assay has been described previously [21]. Briefly, the middle portion of not yet fully-expanded 4th leaves were treated separately with two 10 μl droplets of 10-OPEA (1 mM or 2 mM, dissolved in 5% DMSO and 0.1% Tween 20) or salicylic acid (10 mM or 20 mM, dissolved in 1% or 2% ethanol and 0.1% Tween 20). At 24 h (10-OPEA)/72 h (SA) after treatment, lesions were photographed and digitally assessed using ImageJ software (Image J 1.36b; Wyne Raband, NIH, Bethesda, MD, USA). The assay is illustrated in Additional file 2: Figure S1.

### Protein extraction and Western blot

These methods have been published previously [17, 18].

### qRT-PCR

Leaf samples were collected from 70 days old maize plants in the field, frozen immediately in liquid nitrogen and stored at -80 °C until further use. RNA was isolated from 100 mg of leaf tissue homogenized in liquid nitrogen using TRIzol reagent (ThermoFisher scientific, USA). RNA integrity was assess using formaldehyde denaturing agarose gels, and the yield of quantified using Nanodrop spectrophotometer. For cDNA synthesis, 3 μg of total RNA per sample was used to synthesize cDNA using RevertAid First Strand cDNA Synthesis Kit (ThermoFisher scientific, USA), with oligo (dT) primers. The yield of cDNA was quantified using Nanodrop spectrophotometer, and was further diluted to 40 ng/uL for further use.

From greenhouse-grown plants RNA was extracted from the individual emergent but not yet fully-expanded 4th leaves of the maize plants with RNeasy Plant Mini Kit (Qiagen). Turbo DNA Free Kit (Ambion) was used to purify RNA. 2 μg RNA was reverse transcribed with the Tetro cDNA Synthesis Kit (Bioline).

qRT-PCR was performed in a 20 μl volume [10 μl SYBR Green PCR Master Mix (Applied Biosystems), 1 μl cDNA, 0.5 μl Primer 1 (10 μM), 0.5 μl Primer 2 (10 μM) and 8 μl H_2_O] with iCycler iQ Real-Time PCR Detection System (Bio-Rad). The reaction was run under the following cycle: 10 min at 95 °C, 40 cycles of 15 s 95 °C, 1 min 60 °C. Dissociation analysis was performed after each reaction. The relative expression of the polygalacturonase (*GRMZM2G135763)* gene in different genotypes was calculated by ΔCt method. The Glyceraldehyde-3-phosphate Dehydrogenase (GAPDH) housekeeping gene was used to standardize the results. Primers sequences are provided in Additional file 1: Table S1. These methods have been published previously [17, 18].

## Results

### Gene GRMZM2G135763 encodes a polygalacturonase homolog

The maize gene GRMZM2G135763 was previously identified as a candidate gene for modulation of HR induced by Rp1-D21 [15] as it spanned a SNP marker at 121,288,617 bp on chromosome 7 (based on the maize B73 genome v2 at maizegdb.org) that was highly associated with variation in this trait (Fig. 1a). It encodes five predicted exons and 4 introns (Fig. 1b) which directs production of a protein of 516 amino acids. It shows strong homology to a number of plant polygalacturonases and predicted polygalacturonases with the strongest homologies to genes from other monocots. No other genes in maize have more than a 40% homology at the amino acid level. A search using the Pfam database (http://pfam.xfam.org/) [22] identifies the gene as member of the glycoside hydrolase family 28 with homology between amino acids 68–425, a family that includes polygalacturonases [23]. Additional searches in the CDD (Conserved Domain Database) [24] also identified GRMZM2G135763 as a polygalacturonase. With the important caveat that we have no direct functional confirmation of its polygalacturonase activity, we refer to GRMZM2G135763 as *Zea mays polygalacturonase homolog 1* or *ZmPGH1*.

### *ZmPGH1 suppresses* chemical-induced cell death in maize

We obtained fifteen seeds of UniformMu family UFMu-02561 from Maize Coop Stock Center. These seeds were segregating for the presence of a Mu transposon insertion (mu1029380) ~ 1.8 Kbp upstream of the predicted ATG site of *ZmPGH1* (Fig. 1b) as well as for at least two other Mu insertions. Importantly, the UniformMu families are in a uniform genetic background of the commonly used maize inbred W22 [19] so that progeny of selfed or crossed UniformMu plants only segregate the Mu insertions themselves, while the rest of the genome remains constant.

The seeds were grown out and self-pollinated. From the resulting F_2_ families we identified 5 families homozygous for the mu1029380 insertion and 6 families in which the mu1029380 insertion was absent. We then used quantitative real time PCR (qRT-PCR) to quantify the mRNA levels of *ZmPGH1*in plants homozygous present and absent for insertion mu1029380. Surprisingly, a substantially enhanced level of *ZmPGH1* expression was detected in plants homozygous for mu1029380 compared to plants without the insertion. The four mu1029380 homozygous plants assessed accumulated 1.6-, 2.2-, 4- and 12.7-fold more *ZmPGH1* transcript than plants from the same family that were homozygous absent for the mu1029380 insertion (Fig. 1c). Hence, the mu1029380 insertion causes enhancement of *ZmPGH1*gene expression. Plants homozygous for the mu1029380 insertion were therefore termed *ZmPGH1* over-expressor or ZmPGH1OE plants. F_2_ families lacking the mu1029380 insertion are henceforth referred to as WT families and F_2_ families homozygous for the mu1029380 insertion are referred to as OE families.

**Fig. 2:**
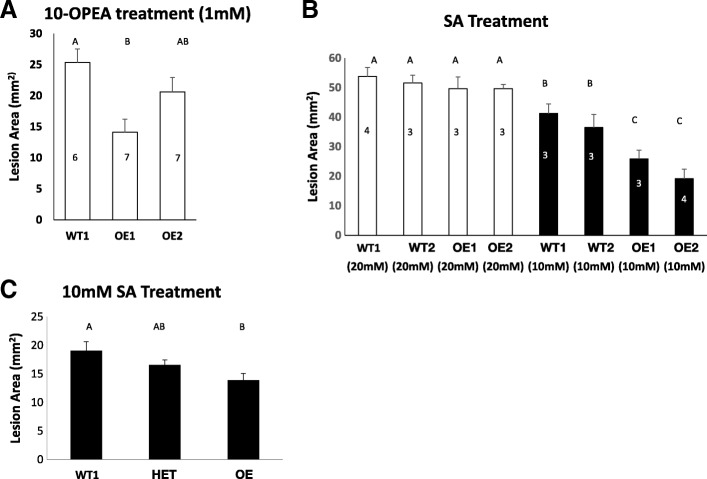
*ZmPGH1* suppresses chemical-induced cell death in maize. (**a**, **b**) The comparisons of average areas (in square millimeter) of 10-OPEA- (**a**) or SA- (**b**) induced lesions (cell death) between families derived from F_2_ segregants from the UFMu-02561 family homozygous for the mu102938 insertion (here termed OE) compared to families derived from segregants homozygous for the absence of the insertion (termed WT). Differently numbered lines (i.e WT1, WT2) indicate individual families. Error bars represent standard error; the numbers of the plants used were shown on the bars. Two lesions were measured on each plant; (**c**) Comparisons of average area (in square millimeter) of 10 mM SA-induced lesions (cell death) among 41 plants from an UFMu-02561 F_2_ family including *7* segregants homozygous absent for the mu1029380 insertion (WT), 22 segregants heterozygous for the mu1029380 insertion (HET) and 12 segregants homozygous for the insertion (OE). Pairwise comparisons for all means were performed with One-Way ANOVA test followed by Tukey-Kramer HSD at 90% confidence limits

Christensen et al. [21] had previously shown that 10-oxo-11-phytoenoic acid (10-OPEA) induced PCD in plant leaves. Treatment with high concentrations of salicylic acid (SA) has also been shown to induce cell death and through the induction of HR-like mechanisms [25]. The emerging (but not fully expanded) 4th leaves from UFMu-02561 WT families and OE families were separately treated with 10-OPEA and salicylic acid (SA). At 1 day-post-treatment, the area of lesions induced by 1 mM 10-OPEA treatment in the WT plants were almost 2-fold greater than the areas in plants of the OE1 family and 20% greater than in plants of the OE2 family (Fig. 2a). Similarly, the lesions induced by 10 mM SA in the WT family plants were 1.5-~ 2-fold greater than those in OE family plants at 3 day-post-treatment (Fig. 2b). These results suggest that ZmPGH1 is a suppressor of plant PCD.

Differences in lesion areas were not observed in wild type and ZmPGH1OE plants treated with 20 mM SA, Fig. 2b). This may result from the higher concentration of the chemicals ‘saturating’ the response of both genotypes, making it impossible to observe differential reactions.

The UFMu-02561 line harbors three known Mu transposons located on chromosomes 1, 7 and 8 and may contain others that have not been characterized. To confirm that the reduced cell death phenotype observed was associated specifically with the presence of the mu1029380 transposon inserted upstream of *ZmPGH1 on* chromosome 7, an F_2_ population segregating for the mu1029380 insertion was created by self-pollinating a plant heterozygous for the mu1029380 insertion. Forty-one plants from this population were assessed using the SA-induced cell death assay and subsequently genotyped by PCR. The population included 7 plants homozygous absent for mu1029380, 22 heterozygous plants and 12 plants homozygous for the mu1029380 insertion. Compared with the plants lacking the mu1029380 insertion, the homozygous mutant plants carrying two copies of the insertion displayed reduced SA-induced cell death areas (Fig. 2c, P < 0.1). The phenotype of the mu1029380 heterozygous plants was intermediate compared to the other two genotypes (Fig. 2c). This demonstrates that the altered cell death phenotype was associated with the mu1029380 insertion and with over-expression of *ZmPGH1*.

### ZmPGH1 suppresses NB-LRR protein-induced HR in *N. benthamiana*

**Fig. 3:**
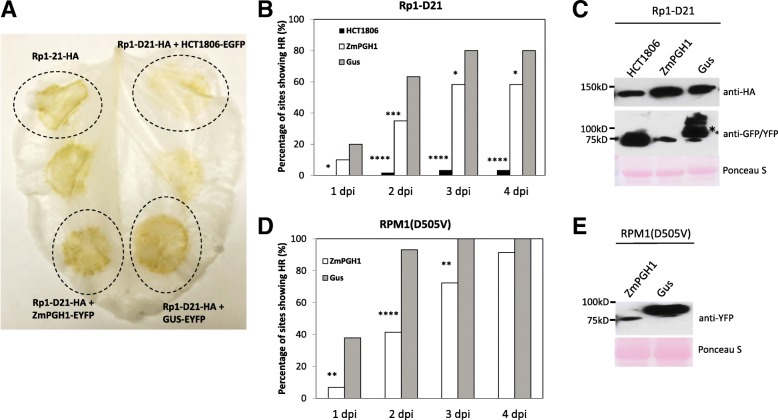
ZmPGH1 protein suppresses NB-LRR protein-induced HR in *N. benthamiana.* (**a**) The HR induced by the transient expression of Rp1-D21-HA and the transient co-expression of HCT1806-EGFP, ZmPGH1-EYFP, or GUS-EYFP with Rp1-D21-HA. The picture was taken at 3 day-post-infiltration and this *N. benthamiana* leaf was bleached with ethanol to make HR more obvious; (**b**, **d**) Percentages of infiltration sites showing the HR induced by the co-expression of HCT1806-EGFP, ZmPGH1-EYFP or GUS-EYFP with Rp1-D21-HA (**b**) or the co-expression of ZmPGH1-EYFP or Gus-EYFP with RPM1(D505V) (**d**). The sites were scored each day post infiltration (dpi); (**c**, **e**) Western blot experiments were performed to detect transiently expressed proteins in *N. benthamiana* leaves. Total proteins were extracted from the infiltrated leaves at 40 h-post-infiltration (**c**, Rp1-D21) or 20 h-post-infiltration (E, RPM1). Anti-HA antibody was used to detect Rp1-D21-HA protein. Anti-GFP was used to detect HCT1806-EGFP, ZmPGH1-EYFP and Gus-EYFP proteins. Equal loading was shown by the red bands of Ponceau S-stained RuBisCO protein. All the experiments shown in this figure were performed at least two times with similar results. . Significant differences from GUS-EYFP are indicated at * *p* < 0.1 ** *p* < 0.05 ***p < 0.01 ****p < 0.001. Significance determined by 2-way ANOVA

To determine whether ZmPGH1 also plays a role in the control of Rp1-D21 induced HR, we used an Agrobacterium-mediated transient expression assay. We previously demonstrated that transient overexpression of Rp1-D21-HA (Rp1-D21 with a C-terminal HA tag) induced HR in *N. benthamiana* leaves [16]. We have also previously shown that co-overexpression of HCT1806-EGFP (hydroxycinnamoyltransferase 1806, GRMZM2G061806, fused with C-terminal enhanced GFP tag) with Rp1-D21-HA can almost completely suppress Rp1-D21-induced HR (Fig. 3a) [16].

In this study, we co-expressed ZmPGH1-EYFP (ZmPGH1 with a C-terminal EYFP tag) with Rp1-D21-HA to determine if ZmPGH1 can affect Rp1-D21-induced HR. As the β-glucuronidase (GUS) reporter gene does not affect Rp1-D21-induced HR [16], a GUS-EYFP construct (GUS gene fused with C-terminal EYFP tag) was employed as a negative control to exclude a possible effect of the EYFP-tag expression vector on HR. Our initial visual observations suggested that the HR induced by the co-expression of Rp1-D21-HA and ZmPGH1-EYFP was weaker than the HR induced by the co-expression of Rp1-D21-HA and Gus-EYFP (Fig. 3a). However, these differences were subtle, so to confirm that ZmPGH1 could suppress Rp1-D21-induced HR, we used a HR dynamic assay, modified from a previously published method [26, 27], to assess the effect of ZmPGH1 on Rp1-D21-induced HR in a quantitative manner.

Three combinations of expression constructs, Rp1-D21/ ZmPGH1, Rp1-D21/HCT, and Rp1-D21/GUS, were co-infiltrated into *N. benthamiana* leaves. Each combination was infiltrated into no fewer than 30 sites in no fewer than 4 leaves. The number of sites displaying HR was assessed every day at the same time for four days. As expected, very few sites co-expressing Rp1-D21 and HCT showed HR, while almost all of sites co-expressing GUS and Rp1-D21 showed HR (Fig. 3b). The proportion of sites co-expressing the combination of ZmPGH1 and Rp1-D21 fell between these extremes, suggesting that ZmPGH1 could partially suppress Rp1-D21-induced HR. Western blots confirmed the expression and the expected molecular weights of the Rp1-D21-HA, HCT1806-EGFP, ZmPGH1-EYFP and Gus-EYFP proteins in *N. benthamiana* leaves (Fig. 3c). Rp1-D21 was detected at similar level when co-expressed with either ZmPGH1-EYFP or GUS-EYFP, suggesting the partially HR suppression caused by ZmPGH1protein is not due to less Rp1-D21-HA protein. Taken together, these results confirmed ZmPGH1 can partially suppress Rp1-D21-induced HR, though not as much as HCT1806 (Fig. 3b).

Several NB-LRR proteins have been reported to induce HR when expressed in *N. benthamiana* leaves [28–32]. RPM1(D505V) is an autoactive mutant allele of Arabidopsis NB-LRR protein RPM1 [29]. We used the HR dynamic assay to determine the effect of ZmPGH1 on RPM1(D505V)-induced HR. The HR induced by RPM1(D505V) was stronger and faster than the HR induced by Rp1-D21 (compare Fig. 3b and d). Compared with the control construct Gus-EYFP, co-expression of ZmPGH1-EYFP partially suppressed RPM1(D505V)-induced HR. We were unable to detect the presence of RPM1(D505V) protein by western blotting, possibly because it rapidly killed the cell in which it was expressed, but we were able to confirm the expression of GUS and the ZmPGH1-EYFP proteins (Fig. 3e). Hence, ZmPGH1 protein could suppress HR induced by multiple NB-LRR proteins.

### Overexpression of ZmPGH1 is associated with suppression of the effects of Rp1-D21 in maize

In order to directly examine the effects of the mu1029380 insertion that caused over-expression of ZmPGH1 on the phenotype conferred by *Rp1-D21*, we combined both these alleles in a uniform genetic background. Since the mu1029380 insertion was in a W22 background, we crossed ZmPGH1OE plants homozygous for this insertion to W22:Rp1-D21; a W22 line into which the Rp1-D21 gene had been introgressed through repeated backcrossing. From this we derived a population in a W22 background in which the mu1029380 insertion was segregating 1:1 homozygous: heterozygous and the *Rp1-D21* gene was also segregating 1:1 heterozygous: absent.

**Fig. 4:**
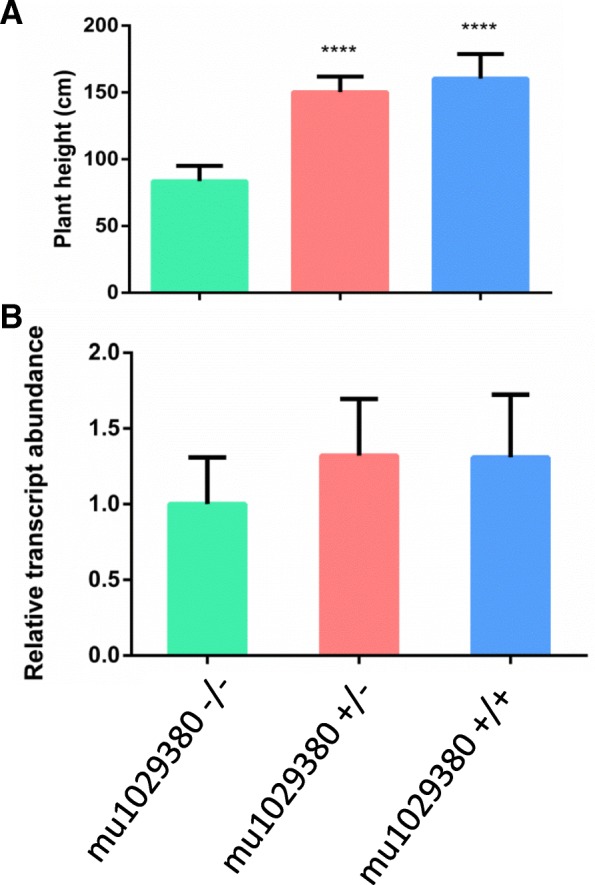
ZmPGH1 suppresses the effects of Rp1-D21 in maize. (**a**). W22 Maize plants heterozygous for Rp1-D21 and homozygous (red bar) or heterozygous (blue bar) for mu1029380 are significantly taller than W22 Maize plants heterozygous for Rp1-D21 that lack mu1029380 (green bar). Data are derived from a replicated field trial in which 13 plants homozygous for mu1029380, 13 plants heterozygous for mu1029380 and 8 plants lacking mu1029380 were measured. **** indicates significant difference from the mu1029380 absent category (p < 0.001). (**b**) Maize plants heterozygous for Rp1-D21 and homozygous (red bar) or heterozygous (blue bar) for mu1029380 express ZmPGH1 at higher levels than maize plants heterozygous for Rp1-D21that lack mu1029380 (green bar). Data are derived from a replicated field trial in which 12 plants homozygous for mu1029380, 12 plants heterozygous for mu1029380 and 8 plants lacking mu1029380 were measured

The W22 line has a mild leaf spotting or ‘flecking’ trait [33] which makes it hard to evaluate the severity of HR induced by Rp1-D21. We have shown previously that the reduction in plant height induced by Rp1-D21 is a reliable indicator of the strength of the Rp1-D21 phenotype [34]. We therefore evaluated this population in replicated field trials, measuring the height of all the segregants carrying Rp1-D21. Plants carrying Rp1-D21 in a heterozygous state and mu1029380 in either a homozygous or heterozygous state were significantly taller at flowering than the W22:Rp1-D21 control (which was also heterozygous for Rp1-D21, Fig. 4a). The expression of *ZmPGH1* was increased in the field grown plants carrying mu1029380 (Fig. 4b) to approximately the same extent as had been previously observed in plants grown in the growth chamber (Fig. 1c), though the differences were not significant at *p* < 0.05 due to relatively high variability associated with field assays. These results suggest that higher expression of *ZmPGH1* suppresses the effects of Rp1-D21 in maize, which is consistent with the results of mapping, transient expression and chemically induced PCD experiments described above.

## Discussion

The cell wall plays a central role in plant-pathogen interactions [35–37]. It is often the first barrier which the pathogen must overcome and is the plant’s first line of defense. Most plant cell walls consist of cellulose fibrils cross-linked with hemicelluloses in a matrix of pectins and lignin. Polygalacturonases catalyze the degradation of pectin in the cell wall via the hydrolytic cleavage of glycosidic bonds [38]. Many plant pathogens produce polygalacturonases to facilitate the breakdown of the cell wall and promote pathogenesis. Plants also produce polygalacturonases. These enzymes are important in fruit ripening and many other developmental process [39]. Several maize polygalacturonases have previously been characterized to some extent [40–42] but, to our knowledge, the *ZmPGH1* (GRMZM2G135763) gene has not been previously investigated. All these previously-characterized polygalacturonases were shown to be strongly expressed in maize pollen where their role was assumed to be in facilitating pollen growth through the stigma. The maize gene expression atlas [43, 44] available at maizegdb.com (validated 1/18/2018) shows that GRMZM2G135763 is expressed throughout development and throughout the plant but that expression is particularly strong in the anthers and developing roots (expression specifically in pollen was not assessed).

ZmPGH1 appears to function as a general suppressor of PCD plants. The enhanced expression of *ZmPGH1* gene in maize suppressed the effects of Rp1-D21 and the cell death caused by SA and 10-OPEA while the transient overexpression of ZmPGH1 in *N. benthamiana* suppressed Rp1-D21- and RPM1(D505V)- induced HR. The measurement of plant height to assess the effect of the Rp1-D21 phenotype was a somewhat indirect measurement, however we have used this approach previously with some success [15, 45]. Height reduction correlated strongly with lesion severity in these studies. We did not observe a consistent difference in lesion severity between plants with and without mu1029380 in the present study. We believe that his was due to the natural and somewhat variable spotting phenotype that we observe in the maize line W22, the line used to create the transposon insertion line, in our environment [33]. This makes it difficult to accurately quantify small differences in spotting caused by expression of *Rp1-D21*.

The Mu transposon insertion mu1029380, ~ 1.8 Kbp upstream of the predicted ATG site of *ZmPGH1* enhanced accumulation of *ZmPGH1* message at least 1.5- fold*.* This expression enhancement may have been caused by the insertion abolishing the binding site of a transcriptional repressor or the Mu transposon might itself drive downstream gene transcription [46]. Robbins et al. [47] characterized a Mu insertion in the 5′ UTR of the *pericarp color 1* gene allele *P1-wr* that increased expression. We have also previously observed a Mu insertion in the 3′ UTR that increased mRNA levels [27]. While the mechanism underlying this increased expression was not unequivocally determined, it appears to be associated with reduced DNA methylation.

In previous studies we demonstrated that two other enzymes predicted to be important in plant cell wall synthesis were also associated with variation in the severity of HR mediated by Rp1-D21. Hydroxycinnamoyltransferase (HCT) and caffeoyl CoA O-methyltransferase (CCoAOMT) catalyze sequential steps in the lignin biosynthesis pathway. Genes encoding these enzymes, *CCoAOMT2* and *HCT1806/HCT4918* (two tightly linked and highly homologous HCT genes) were both initially associated with variation in Rp1-D21-mediated HR in the same mapping study in which *ZmPGH1* was identified [15]. We demonstrated that both were able to suppress Rp1-D21-mediated HR and that they physically interacted with Rp1-D21 and with each other to suppress its autoactivation [16, 17]. In the case of CCoAOMT2 we also showed that it affected HR mediated by RPM1(D505V), though to a lesser extent [27] and that the enzyme was associated with variation in resistance to at least two foliar fungal necrotrophic maize diseases, SLB and gray leaf spot [27]. In this study we implicate a third cell wall enzyme in the control of HR and PCD. Of note also is the fact that the expression of all three genes, *ZmPGH1, HCT1806/HCT4918* and *CCoAOMT2*, were induced by Rp1-D21-induced HR (1.9-, 2.2- and 296-fold respectively) and by treatment with 10-OPEA (1.8-, 4.5- and 45.8 fold respectively) which suggests they are associated with a response to PCD [15, 21].

Polygalacturonases and polygalacturonase inhibiting proteins as well as other enzymes involved in pectin degradation have previously been implicated in plant disease resistance and response [48–54], as have lignin and the phenylpropanoid pathway branch leading to lignin biosynthesis [17, 35–37]. Different plant PCD responses have different effects on the cell wall. In some cases, such as in abcission zones and in leaf formation in the lace plant, the cell wall is completely degraded [55, 56]. In other cases, such as during xylem vessel formation it is partially degraded [57]. Histological studies in various plants suggest that the cell wall appears to remain largely intact during HR [58, 59] though lignification or thickening are often observed [60]. While the mechanism by which ZmPGH1 inhibits PCD processes is not clear it seems likely that its PCD effect may be mediated through its effect on the cell wall. We plan to investigate this possibility in future work.

## Conclusions

*ZmPGH1* functions as a suppressor of programmed cell death induced by at least two autoactive NLR proteins and by two chemical inducers. These findings deepen our understanding of the role of the cell wall in mediting disease resistance and the defense response, including control of the HR in plants.

## Additional files

Additional file 1: **Table S1.** PCR primers used in this study (PDF 213 kb)

Additional file 2: **Figure S1.** Chemical-Induced Cell Death Assay. This assay has been described previously [21]. A. The middle portion of individual emergent but not yet fully-expanded 4th leaves (indicated by yellow circle) are separately treated with two 10 μl droplets of 10-OPEA (1 mM or 2 mM, dissolved in 5% DMSO and 0.1% Tween 20) or salicylic acid (10 mM or 20 mM, dissolved in 1% or 2% ethanol and 0.1% Tween 20). B. At 24 h (10-OPEA)/72 h (SA) post treatment, lesion areas are photographed and digitally measured using ImageJ software (Image J 1.36b; Wyne Raband, NIH, Bethesda, MD, USA). (PPTX 1201 kb)

## Data Availability

Data and materials are available on request from the corresponding author. This study does not use any large datasets so no supplementary data has been deposited in public repositories.

## Abbreviations

CCoAOMT: Caffeoyl CoA O-methyltransferase
ETI: Effector-triggered immunity
Fig: Figure
GWAS: Genome-wide association study
HCT: Hydroxycinnamoyltransferase
HR: Hypersensitive Response
MAMP: Microbe-associated molecular pattern
MTI: Mamp-triggered immunity
NAM: Nested association mapping
NLR: Nucleotide-binding site, leucine-rich repeat
PRR: Pattern recognition receptor
R-protein: Resistance protein
SNP: Single nucleotide polymorphism
