# Supplementary material for: A maize polygalacturonase functions as a suppressor of programmed cell death in plants
Source: BMC Plant Biol. 2019 Jul 15;19:310. doi: 10.1186/s12870-019-1897-5 (PMC6628502; doi:10.1186/s12870-019-1897-5)
Supplement: Supplementary file 1 — Table S1. PCR primers used in this study (PDF 213 kb) [file 12870_2019_1897_MOESM1_ESM.pdf]

Table S1. PCR primers used in this study.

| Primer Name | Sequence (5'-3') | Notes |
|---|---|---|
| Polygalacturonase**cDNA cloning primers:** | | |
| PolygalacturonaseattB 1 | *GGGGACAAGTTTGTACAAAAAAGCAGGCTCC*ATGCTGGAGGCCACCG | Parts in italics indicate sequences of attB1 and attB2 adaptors. attB adaptors used to clone the products into pDONR207 vector |
| PolygalacturonaseattB 2 | *GGGGACCACTTTGTACAAGAAAGCTGGGTG*GAAACTGTAGTCGATATCAGAG | |
| **qRT-PCR Primers:** | | |
| Polygalacturonase-RT-F | CCCACCATCGGGAATATCTCTTACA | |
| Polygalacturonase-RT-R | GCACTGGAAAACATGGTGCTTCTTG | |
| GAPDH-RT-F | ATCAACGGCTTCGGAAGGAT | |
| GAPDH-RT-R | CCGTGGACGGTGTCGTACTT | |
| **Mu Insertion Genotyping Primers** | | |
| Polygalacturonase-Mu-F | TAGTACTTCCAACACGCGAACG | |
| Polygalacturonase-Mu-R | CGTCCACATCGTCTTCTCTGAT | |
| TIR6 | AGAGAAGCCAACGCCAWCGCCTCYATTTCGTC | The TIR6 primer was used as Mu TIR specific prime [19] |
