# Supplementary figures and images for: A maize polygalacturonase functions as a suppressor of programmed cell death in plants
Source: BMC Plant Biol. 2019 Jul 15;19:310. doi: 10.1186/s12870-019-1897-5 (PMC6628502; doi:10.1186/s12870-019-1897-5)

## Slide 1
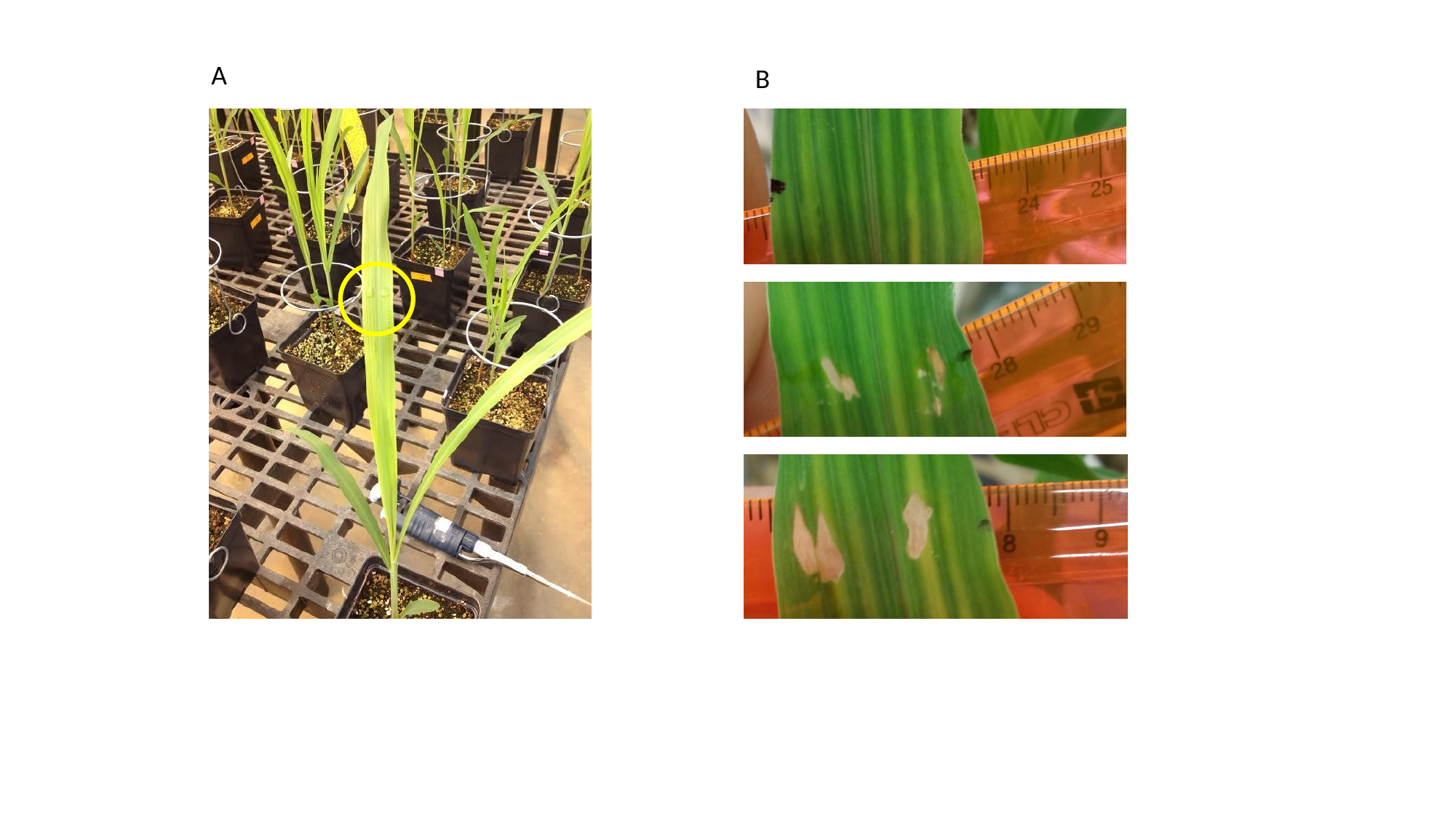

A
B

Supplement: Supplementary file 2 — Figure S1. Chemical-Induced Cell Death Assay. This assay has been described previously [21]. A. The middle portion of individual emergent but not yet fully-expanded 4th leaves (indicated by yellow circle) are separately treated with two 10 μl droplets of 10-OPEA (1 mM or 2 mM, dissolved in 5% DMSO and 0.1% Tween 20) or salicylic acid (10 mM or 20 mM, dissolved in 1% or 2% ethanol and 0.1% Tween 20). B. At 24 h (10-OPEA)/72 h (SA) post treatment, lesion areas are photographed and digitally measured using ImageJ software (Image J 1.36b; Wyne Raband, NIH, Bethesda, MD, USA). (PPTX 1201 kb) [file 12870_2019_1897_MOESM2_ESM.pptx]
